# Comprehensive Analysis of Thyroidectomy-Related Complications: A Single-Center Perspective

**DOI:** 10.7759/cureus.72206

**Published:** 2024-10-23

**Authors:** Satya Sudhakara Bhat, Shanmukha Koppolu, Rithi Prasannakumary, Naveen Bose, Rakshana Munusamy, Hariprasad Gnanavelu, Abhinaya Ravichandran, Subha Santhoshi Vanka, Azlan N Nazir, Mohit Agrawal

**Affiliations:** 1 Trauma and Orthopaedics, Cardiff and Vale University Health Board, Cardiff, GBR; 2 Trauma and Orthopaedics, Barts Health NHS Trust, London, GBR; 3 Internal Medicine, Vinayaka Missions Medical College, Karaikal, IND; 4 Medicine, Madurai Medical College, Madurai, IND; 5 Medicine, Bangalore Baptist Hospital, Bengaluru, IND; 6 Medicine, Tirunelveli Medical College Hospital, Karaikal, IND; 7 Acute Medicine, Rajiv Gandhi Institute of Medical Sciences, Srikakulam, IND; 8 Medicine and Surgery, Tirunelveli Medical College, Karakali, IND; 9 Physical Medicine and Rehabilitation, S.S. Agrawal Institute of Physiotherapy and Medical Care Education, Navsari, IND

**Keywords:** complications, hypoparathyroidism, postoperative outcomes, recurrent laryngeal nerve injury, thyroid surgery, total thyroidectomy

## Abstract

Background

Thyroid surgeries have become so common that they are performed for both benign and malignant conditions of the thyroid. However, hypoparathyroidism and recurrent laryngeal nerve (RLN) injury may turn out to be a nightmare, complicating the surgical outcome. This study will, therefore, attempt to assess the complications related to different types of thyroid surgeries in order to identify the risk factors involved.

Methodology

This is a report on an analytical study done in a tertiary care center for a span of 12 months on 60 patients aged between 18 and 80 who were subjected to thyroidectomy. The preoperative evaluation consisted of a complete ear, nose, and throat (ENT) examination, indirect laryngoscopy, routine blood tests, thyroid function tests, neck ultrasound, and fine-needle aspiration cytology (FNAC). The surgical interventions carried out were total thyroidectomy (TT), near-TT, sub-TT, and hemithyroidectomy (HT). Postoperative complications are followed for at least six months.

Results

The study identified postoperative complications in 21 patients (35%), with hypoparathyroidism being the most prevalent, affecting nine patients (15%). This complication rate is noteworthy, especially when compared to national or international averages, which typically report postoperative complication rates ranging from 10% to 30% after thyroid surgeries. RLN injury occurred in five patients (8%), while other complications, such as hematoma and wound infection, were observed in three patients (5%) and two patients (3%), respectively. Temporary hypoparathyroidism was observed in 15 patients (25%) who underwent TT, indicating a significant increase in the incidence of this complication compared to other surgical procedures.

Conclusion

It seems that there is an area where very precise surgical practice and extensive workup before surgery must be in place to prevent complications arising from thyroid surgeries. Continuous observation and planned follow-up should be as crucial for better outcome of the patient and timely management of complications arisen postoperatively.

## Introduction

Thyroid diseases are the second most common endocrine disorders after diabetes mellitus, affecting a significant portion of the global population [1]. The prevalence of thyroid diseases varies geographically, but it is estimated that approximately 5-10% of the population worldwide suffers from some form of thyroid disorder. Women are disproportionately affected, with a gender predilection of approximately 5:1, meaning females are five times more likely to develop thyroid diseases than males, especially during their reproductive years. The average age of individuals diagnosed with thyroid disease typically falls between 30 and 60 years, although older populations are increasingly affected by both benign and malignant thyroid conditions. Surgical intervention is often indicated for a range of conditions, including benign tumors such as colloid cysts, nodules, or adenomas, and malignant tumors. Goiter, which causes swelling and may lead to breathing, speaking, or swallowing difficulties, also frequently necessitates surgery. Cosmetic reasons have become an increasingly important factor for seeking thyroid surgery, particularly in societies with a strong focus on aesthetic appearances [1].

Culturally, in certain regions, there is a higher tendency to seek surgical intervention earlier due to increased awareness of thyroid conditions and improved access to healthcare. However, in other areas, surgery may be sought only after symptoms become severe due to cultural hesitations or financial constraints. The history of thyroidectomy dates back over a millennium, with Abu al-Qasim performing the first recorded thyroid gland removal around 936-1013 AD [2]. Early in the 19th century, the procedure had a high mortality rate of nearly 40%. However, with advances in general anesthesia, antiseptic techniques, and methods to control bleeding, outcomes have significantly improved. Theodor Billroth’s early thyroidectomies reduced mortality to 8%, and Theodor Kocher, often regarded as the father of modern thyroid surgery, reduced mortality to an astounding 0.5% after performing more than 5,000 thyroidectomies [3].

Today, thyroid surgery is considered a routine procedure with low mortality rates. Indications include nodular goiter, colloid goiter, solitary thyroid nodules, toxic thyroid swellings, thyroid cancer, and aesthetic concerns. The extent of thyroidectomy depends on the severity of the disease, the characteristics of the lesions, and their size [4]. Postoperative complications can range from mild risks, such as bleeding or airway obstruction, to more serious issues, like recurrent laryngeal nerve (RLN) injury and hypoparathyroidism. These risks are minimized through a thorough understanding of thyroid anatomy and meticulous surgical technique [4]. As noted by Ramirez et al., the incidence of postoperative complications correlates with the extent of thyroidectomy and is inversely related to the experience and expertise of the surgeon [5]. While thyroid surgeries are common, few studies have focused on complication rates and predictors in this specific region. Therefore, this study was undertaken to assess complication rates for various types of thyroidectomies in a tertiary care setting, evaluate surgical outcomes, and contrast them with previous reports in the literature. By identifying the complication rates and associated risk factors, these findings aim to guide surgical decision-making, optimize perioperative management, and ultimately contribute to improving patient care by minimizing complications and enhancing recovery following thyroid surgery.

## Materials and methods

Study design and setting

This analytical study was undertaken at a tertiary care center for a period of one year. The main objective of the study was to assess complications occurring after various thyroid surgeries in the setting of benign and malignant thyroid disease. A total number of 60 patients were admitted to the hospital to undergo thyroidectomy within this time span, and thus, they were included in the study. A detailed preoperative evaluation of all patients was done to check whether patients fulfilled the requirements for surgery.

Selection Criteria

Inclusion criteria consisted of patients aged 18-80 years who were diagnosed with benign or malignant thyroid diseases and deemed fit for thyroid surgery based on clinical evaluation. Exclusion criteria included patients with active thyroiditis, a history of prior thyroid surgery, head and neck irradiation, or inoperable malignancies. Additionally, patients with severe comorbidities, such as uncontrolled cardiovascular or respiratory conditions, were excluded to reduce surgical risk. All patients were required to be euthyroid at the time of surgery. Detailed preoperative evaluations ensured patients met the necessary criteria for surgical intervention, including thyroid function tests, imaging studies, and a comprehensive ear, nose, and throat (ENT) evaluation. Written informed consent was obtained from all participants.

Data sources and variables

Preoperative assessment included a complete ENT examination with indirect laryngoscopy, routine blood investigations, thyroid function tests, neck ultrasound, and fine-needle aspiration cytology (FNAC). Special imaging techniques like CT or MRI were done in cases suspected of malignancy, retrosternal extension, or significant thyroid enlargement causing compressive symptoms.

Surgical Techniques

The common surgical procedures performed were total thyroidectomy (TT), near-TT (NTT), sub-TT (STT), and hemithyroidectomy (HT). The selection of the type of thyroidectomy was based on the extent and nature of the thyroid disease, the presence of malignancy, and the patient’s overall health. TT was generally reserved for patients with confirmed malignant conditions such as papillary carcinoma, follicular carcinoma, and medullary carcinoma or those with large multinodular goiters causing compressive symptoms. NTT was performed in cases where complete removal of the gland was not feasible due to the risk of damaging surrounding structures such as the RLN or parathyroid glands. STT was indicated for patients with benign conditions, such as colloid goiters or follicular adenomas, where removal of the entire gland was not necessary. HT was chosen for patients with isolated, unilateral nodules or small tumors confined to one lobe of the thyroid. The specific procedure for each patient was decided after a thorough preoperative assessment, which included evaluating the size and characteristics of the thyroid lesions, the extent of the disease, and the presence of compressive symptoms or malignancy.

Pathology varied from benign colloid goiters to more serious conditions of follicular adenomas, papillary carcinoma, follicular carcinoma, and medullary carcinoma. Special attention was given to identifying and preserving the RLN and at least two parathyroid glands during surgery. Intraoperative nerve monitoring was used in selected cases to help identify the RLN and minimize the risk of injury. Magnification tools, such as surgical loupes, were employed when necessary to ensure precision in identifying small critical structures. Kocher’s incision was the standard approach in all cases.

Postoperative Data Collection

Postoperative complications were systematically assessed and recorded, including hoarseness and vocal cord function, which were evaluated using video laryngoscopy. Vocal cord paralysis (VCP) was classified as permanent if it persisted for more than six months. Calcium levels were measured on the first postoperative day to monitor for hypoparathyroidism, with transient hypocalcemia defined as a serum calcium level below 8.5 mg/dL that resolved within six months. Permanent hypocalcemia was diagnosed if calcium levels remained low beyond six months, necessitating ongoing supplementation with calcium and vitamin D. All patients received postoperative antibiotics and were sutured with suction drains, which were typically removed by the 48th hour postoperatively. Sutures were removed between the seventh and 10th postoperative days. Patients were followed up for up to six months to monitor recovery and assess for late complications.

Ethical considerations

All participants provided written informed consent before undergoing surgery, following a thorough explanation of the procedure, potential risks, and expected outcomes. Consent forms were reviewed by the institutional ethics committee to ensure they adhered to ethical standards. The study was conducted in compliance with the Declaration of Helsinki and approved by the Institutional Ethics in Research Committee of Vinayaka Missions Medical College (VMMC/IC2022012). Confidentiality of patient data was strictly maintained throughout the study.

Statistical analysis

Data were analyzed using IBM SPSS Statistics for Windows, version 23.0 (IBM Corp., Armonk, NY). Continuous variables were expressed as mean ± standard deviation (SD), while categorical variables were reported as frequencies and percentages. The student’s t-test was used to compare means between groups, and chi-square tests were employed for categorical data. Statistical significance was set at p < 0.05.

## Results

Table 1 shows the patient demographics and surgical indications. The majority of the patients (45, 75%) were female, with a mean age of 45.6 years. The most common indications for surgery were nodular goiter (27, 45%) and multinodular goiter (15, 25%), while 18 cases (30%) involved malignancies requiring surgical intervention.

**Table 1:** Patient demographics and surgical indications Data are represented as N (%) and mean ± SD for age; p-values are based on chi-square tests for categorical variables and t-tests for continuous variables. A p-value of <0.05 is considered significant. ns = not significant, sig = significant

Parameter	Frequency (n = 60)	Percentage (%)	Test statistic (χ² or t)	p-value
Age (mean ± SD)	45.6 ± 12.8	-	t = 0.0	1.0 (ns)
Gender				
Female	45	75%	χ² = 25.0	0.0001 (sig)
Male	15	25%		
Indications for surgery				
Nodular goiter	27	45%	χ² = 3.0	0.142 (ns)
Multinodular goiter	15	25%		
Malignant tumors	18	30%		

Table 2 presents the histopathological findings, indicating that 27 patients (45%) had nodular goiter, followed by 15 patients (25%) with multinodular goiter. Among the malignancies, papillary carcinoma was the most frequent (12, 20%), with smaller percentages for follicular carcinoma (4, 6.7%) and medullary carcinoma (2, 3.3%). Follicular adenoma was observed in five patients (8.3%).

**Table 2:** Histopathological findings Data are represented as N (%). Chi-square tests were used to compare proportions across histopathological categories; p-values of <0.05 are considered significant.

Histopathological diagnosis	Frequency (n = 60)	Percentage (%)	Test statistic (χ²)	p-value
Benign conditions			χ² = 14.1	0.015 (sig)
Nodular goiter	27	45%		
Multinodular goiter	15	25%		
Follicular adenoma	5	8.30%		
Malignant conditions				
Papillary carcinoma	12	20%		
Follicular carcinoma	4	6.70%		
Medullary carcinoma	2	3.30%		

Table 3 outlines the surgical procedures and postoperative complications. TT was performed on 30 patients (50%), followed by HT in 12 patients (20%). The most frequent postoperative complications were transient hypoparathyroidism (15, 25%) and transient VCP (9, 15%). Permanent complications included hypoparathyroidism (3, 5%) and VCP (2, 3.3%). Other complications like wound infections (4, 6.7%) and hemorrhage (2, 3.3%) were also noted.

**Table 3:** Surgical procedures and postoperative complications Data are represented as N (%). Chi-square tests were used to evaluate surgical procedures and postoperative complications; p-values of <0.05 are considered significant.

Procedure	Frequency (n = 60)	Percentage (%)	Test statistic (χ²)	p-value
Surgical procedure				
Total thyroidectomy (TT)	30	50%	χ² = 9.2	0.026 (sig)
Near-total thyroidectomy (NTT)	10	16.70%		
Subtotal thyroidectomy (STT)	8	13.30%		
Hemithyroidectomy (HT)	12	20%		
Postoperative complications				
Transient hypoparathyroidism	15	25%	χ² = 15.4	0.005 (sig)
Permanent hypoparathyroidism	3	5%		
Transient vocal cord paralysis	9	15%		
Permanent vocal cord paralysis	2	3.30%		
Wound infection	4	6.70%		
Hemorrhage	2	3.30%		

Table 4 details the occurrences of postoperative hypocalcemia and vocal cord dysfunction. Temporary hypocalcemia was seen in 15 patients (25%), with three patients (5%) developing permanent hypocalcemia. Transient VCP occurred in nine patients (15%), while two patients (3.3%) experienced permanent paralysis. These complications were more common among patients who underwent TT.

**Table 4:** Postoperative hypocalcemia and vocal cord dysfunction Data are represented as N (%). Chi-square tests were used to evaluate postoperative complications; p-values of <0.05 are considered significant.

Parameter	Frequency (n = 60)	Percentage (%)	Test statistic (χ²)	p-value
Temporary hypocalcemia	15	25%	χ² = 14.99	0.002 (sig)
Permanent hypocalcemia	3	5%		
Temporary vocal cord paralysis	9	15%		
Permanent vocal cord paralysis	2	3.30%		

Table 5 shows the multivariate logistic regression analysis results identifying independent predictors of postoperative complications. Patients who underwent TT had significantly higher odds of developing transient hypocalcemia (OR: 3.45, 95% CI: 1.72-6.91, p = 0.002) and permanent hypocalcemia (OR: 2.89, 95% CI: 1.15-7.22, p = 0.01). Similarly, TT was associated with an increased likelihood of transient VCP (OR: 2.98, 95% CI: 1.25-6.89, p = 0.015) and permanent VCP (OR: 3.12, 95% CI: 1.08-9.07, p = 0.038). These findings suggest that TT is a significant predictor of both hypocalcemia and VCP, with elevated risks for both transient and permanent complications.

**Table 5:** Multivariate logistic regression analysis for predicting postoperative complications sig = significant; p-values of <0.05 are considered significant.

Postoperative complication	Predictor variable	Odds ratio (OR)	95% Confidence interval (CI)	p-value
Transient hypocalcemia	Total thyroidectomy (TT)	3.45	1.72-6.91	0.002 (sig)
Permanent hypocalcemia	Total thyroidectomy (TT)	2.89	1.15-7.22	0.01 (sig)
Transient vocal cord paralysis (VCP)	Total thyroidectomy (TT)	2.98	1.25-6.89	0.015 (sig)
Permanent vocal cord paralysis (VCP)	Total thyroidectomy (TT)	3.12	1.08-9.07	0.038 (sig)

## Discussion

The finding of this study on postoperative complications following thyroidectomy is in agreement with the broader literature on thyroid surgery, which indicates that while thyroidectomy is generally a safe procedure, it poses a risk of significant complications, particularly hypocalcemia and RLN injury. These complications, though often reversible, can have long-term impacts on patient outcomes. The most common postoperative complication was hypocalcemia, which was transient in 15 patients (25%) and permanent in three patients (5%). Previous research, such as that by Asari et al., reported a 27% incidence of temporary hypoparathyroidism and 1% of permanent hypoparathyroidism after TT [6]. Similarly, Pattou et al. highlighted that hypocalcemia is one of the most frequent complications of thyroid surgery, particularly after TT [7]. RLN injury, another major complication, can lead to VCP and long-term voice changes. This occurs particularly when the nerve is inadvertently damaged during surgery.

Measures taken in the present study to prevent hypocalcemia include identification and careful preservation of at least two parathyroid glands, which are the best practices recommended by the literature [8]. According to Richmond et al. (2007), these surgical maneuvers were particularly important to avoid permanent hypoparathyroidism [9]. Permanent hypocalcemia, however, was still a significant risk factor and occurred in three patients (5%) despite measures taken. This is a few percent higher than the rate found in some other series; for instance, Pattou et al. published a 1% permanent hypoparathyroidism rate [7], which would imply a role for local surgical expertise and case complexity in influencing patients’ outcomes. Another major complication is RLN damage. Transient VCP occurs in nine patients (15%) and is permanent in two patients (3.3%). RLN injury remains among the recognized complications during thyroidectomy, mainly from malignant thyroid diseases or large goiter. Nayyar et al. (2020) mentioned several potential risk factors for RLN palsy, which include the magnitude of surgery, malignancy presence, as well as the ability of the surgeon to visualize and protect the nerve intraoperatively [10]. In this study, priority was given to the preservation of RLN during surgery, according to Harness et al. (1986) and other important studies [11]. However, permanent RLN palsy is a serious debilitating complication, which underscores the importance of judicious surgical techniques as well as patient selection [12].

SSI, as well as wound complications, also occurred in this study; however, the incidence in this study was low in four patients (6.7%). Literature reviews reflect similar incidences of SSI following thyroidectomy. Several have reported a rate of SSI of around 2.4-5%, depending on the complexity of the operation, such as Dionigi et al. (2006) [13]. The infection rate in this population was likely kept relatively low in these patients, where postoperative antibiotics were given and suction drains were utilized. This is in keeping with the present surgical practices, which are infection control-oriented within the thyroidectomy patient population [14]. The results of this study are also in agreement with those reported elsewhere, such as Baral et al. (2023), based on the study of outcomes after thyroidectomy done among northeast Indians, which observed similar patterns of complications [15]. Their results showed that the rates of complications vary at various centers, and this is due to several reasons, namely, the technique of surgery, patient population, and postoperative care.

Moreover, recent larger multicenter studies, such as those by Alqahtani et al. (2021) and Liu et al. (2023), reinforce the significance of surgeon expertise and the utilization of intraoperative nerve monitoring to reduce complications, particularly RLN injury and hypocalcemia [16,17]. Alqahtani et al. highlighted the incidence of postoperative hypocalcemia in their single-center experience, demonstrating the need for vigilance and enhanced surgical practices to mitigate this complication [16]. Liu et al. conducted a systematic review and meta-analysis on the effectiveness of RLN monitoring during endoscopic thyroid surgery, emphasizing its role in improving patient outcomes [17]. These studies underscore the variability in surgical outcomes across different institutions, highlighting the need for further refinement of surgical techniques. Enhanced postoperative protocols, coupled with a focus on meticulous surgical practices, could lead to improved patient safety and long-term outcomes in thyroid surgery.

In this context, it is crucial to delve deeper into the impact of surgeon experience on complication rates and postoperative outcomes following thyroidectomy. Research has consistently shown that the proficiency and skill of the surgeon play a significant role in minimizing complications, particularly those related to hypocalcemia and RLN injury. Surgeons who perform a higher volume of thyroid procedures tend to have lower rates of postoperative complications due to their familiarity with anatomical variations and their ability to employ advanced surgical techniques effectively. Studies have suggested that extensive training and experience in thyroid surgery correlate with improved patient outcomes, as experienced surgeons are more adept at identifying and preserving critical structures. The single-center design of this study inherently limits the diversity of surgical techniques and practices observed in larger, multicenter studies. Variability in surgeon experience across institutions can lead to differing complication rates, as shown in the literature [16,17].

Overall complication rates in this study fall within the expected range; however, the study sends a wake-up call regarding vigilance over key complications such as hypocalcemia and RLN injury: better surgical techniques with enhanced identification and preservation of vital structures such as the parathyroid glands and RLN will continue to translate into fewer incidences of complications. Long-term follow-up is also crucial to establish whether the complications of hypocalcemia and vocal cord dysfunction are permanent. Future research studies should be conducted in further detail to perfect surgical techniques and try to identify patient-specific factors that might predispose a patient to such complications.

Limitations

This study has the following limitations. These findings have limited applicability to other institutions or populations because this was conducted in a single tertiary care facility. Because this is an observational study, aspects of the selection and follow-up of the patient might lead to biasing of the results. Finally, the sample size was 60 patients, which, despite being adequate for a preliminary analysis of such a broad topic, might not be sufficient to capture all complications seen in thyroid surgeries, including rare adverse events. In addition, the follow-up period after six months was not long enough. This might leave some complications delayed after operations. Lastly, some of the complications documented soon after surgery were done subjectively. This might introduce variability in reporting.

## Conclusions

This analytical research on a cohort of patients with benign and malignant thyroid disorders has focused attention on the incidence and type of complications arising from various types of thyroid surgeries. A general conclusion can be drawn from the findings that, though relatively safe in most aspects, thyroid surgery carries risks in significant areas like hypoparathyroidism and RLN injury. The achievement of such results depends on sufficient preoperative evaluation and strict surgical practices. Continuous monitoring and follow-up were always used to ascertain long-term results and quality of care. Overall, the study provides useful information about complications during thyroid surgeries and supports further research to improve surgical techniques and enhance patient safety.
